# Thromboxane A2 receptor (TBXA2R) is a potent survival factor for triple negative breast cancers (TNBCs)

**DOI:** 10.18632/oncotarget.10969

**Published:** 2016-07-30

**Authors:** Katy Orr, Niamh E. Buckley, Paula Haddock, Colin James, Jean-Luc Parent, Stephen McQuaid, Paul B. Mullan

**Affiliations:** ^1^ Centre for Cancer Research and Cell Biology, Queen's University Belfast, Belfast, UK; ^2^ Université de Sherbrooke, Sherbrooke, Canada

**Keywords:** TBXA2R, TNBC, BRCA1, cell survival, predictive marker

## Abstract

Triple Negative Breast Cancer (TNBC) is defined by the lack of ERα, PR expression and HER2 overexpression and is the breast cancer subtype with the poorest clinical outcomes. Our aim was to identify genes driving TNBC proliferation and/or survival which could represent novel therapeutic targets.

We performed microarray profiling of primary TNBCs and generated differential genelists based on clinical outcomes following the chemotherapy regimen FEC (5-Fluorouracil/Epirubicin/Cyclophosphamide -‘good’ outcome no relapse > 3 years; ‘poor’ outcome relapse < 3 years). Elevated expression of thromboxane A2 receptor (TBXA2R) was observed in ‘good’ outcome TNBCs. TBXA2R expression was higher specifically in TNBC cell lines and TBXA2R knockdowns consistently showed dramatic cell killing in TNBC cells. TBXA2R mRNA and promoter activities were up-regulated following BRCA1 knockdown, with c-Myc being required for BRCA1-mediated transcriptional repression.

We demonstrated that TBXA2R enhanced TNBC cell migration, invasion and activated Rho signalling, phenotypes which could be reversed using Rho-associated Kinase (ROCK) inhibitors. TBXA2R also protected TNBC cells from DNA damage by negatively regulating reactive oxygen species levels. In summary, TBXA2R is a novel breast cancer-associated gene required for the survival and migratory behaviour of a subset of TNBCs and could provide opportunities to develop novel, more effective treatments.

## INTRODUCTION

Breast cancer research has been revolutionised over the past decade by the advent of gene expression profiling, which at the molecular level, has subdivided breast cancers into five ‘intrinsic subtypes’ (Luminal A and Luminal B, basal-like, HER2 -enriched and a normal breast-like group) [1, 2]. Although molecular profiling of breast tumours has provided valuable insights into the biological behaviour of different subtypes of breast cancer, in the clinical/pathology setting classification of breast cancer is routinely based on IHC staining for ERα, PR and HER2. Triple negative breast cancers (TNBCs), which account for approximately 15% of all breast cancers, are characterised by a lack of ERα, PR expression and HER2 overexpression, and although the majority of TNBCs share similar gene expression profiles with Basal-Like Breast Cancers (BLBCs), there is not complete overlap between the two groups [3]. The TNBC subtype is associated with high mortality rates due to both the aggressive nature of the disease and limited treatment options available. Lacking ERα expression and HER2 receptor amplification/overexpression, TNBC patients do not benefit from either hormonal or HER2-targeting therapies, and as a result, the risk of relapse is 30–40%, compared to less than 25% in ERα positive patients treated with endocrine therapies [4]. Since there are no targeted treatments available for TNBC, these patients are entirely reliant on chemotherapy and the current standard of care is to treat with a combination of DNA damaging agents, most notably the FEC regimen [5].

Whilst some TNBC patients relapse rapidly following primary chemotherapy, many patients achieve a complete pathologic response (pCR) and have a favourable prognosis [6–8]. However, patients with residual disease have a short overall survival and very poor outcomes, with one study reporting a median survival of only 6 months following manifestation of distant metastasis [9]. A further obstacle to treatment of TNBCs is the highly heterogeneous nature of this disease. Numerous attempts have recently been made to sub-classify TNBC in order to identify novel (i) prognostic markers, (ii) predictive markers of chemotherapy responses and (iii) potential therapeutic targets [10, 11] and as many as 10 different subtypes of TNBC have now been described. This highlights the importance of identifying novel therapeutic targets for TNBC patients and a number of avenues of research are currently being explored such as PIK3/AKT and mTOR inhibitors, alongside biomarkers to stratify which subpopulations of TNBCs are most likely to respond to them [12, 13].

The thromboxane A2 receptor (TBXA2R) is a seven-transmembrane domain-spanning G-protein coupled receptor (GPCR) predominantly expressed in platelets and involved in platelet activation and aggregation, but can activate a multitude of signalling cascades and thus regulate a diverse range of cellular processes such as vasoconstriction of smooth muscle cells, inflammatory responses, cell adhesion and motility, proliferation and cell survival [14]. The oncogenic role of TBXA2R in lung cancer has been particularly well studied in which it enhances cell proliferation, migration and invasion, and thromboxane A2 synthase (TBXAS) inhibits apoptosis *via* negative regulation of reactive oxygen species (ROS) [15–18]. High expression levels of TBXA2R have also been observed in bladder cancer and prostate cancer cell line models leading to increased migratory capacity [19–21]. Thromboxane production has been shown to be increased in human mammary carcinomas in comparison to matched normal breast tissue, and correlated with increased tumour size and metastatic potential as well as absence of ERα/PR [22]. Additionally, analysis of TBXA2R mRNA levels in 120 human breast tumours and 32 non-cancerous mammary tissues showed higher levels of TBXA2R transcript were significantly associated with higher grade tumours and shorter disease free survival [23]. Despite the indications that thromboxane signalling is associated with poor prognosis in breast cancer, few studies have investigated the functional role of this pathway in breast cancer.

This current study shows that TBXA2R is highly expressed specifically in TNBC cell line models and loss of expression causes a dramatic decrease in not only cell viability and proliferation but also cell migration and invasion. We have also shown for the first time that TBXA2R is transcriptionally repressed by BRCA1 (a tumour suppressor often mutated or down-regulated in TNBC), providing a potential mechanism by which TBXA2R is up-regulated in TNBC/BLBCs. We have shown that TBXA2R may promote oncogenesis *via* the Rho/ROCK pathway and evidence is presented for ROCK inhibition as a potential treatment option for TBXA2R over-expressing TNBCs. Finally, TBXA2R is indicated as a negative regulator of ROS and a potential predictive marker of chemotherapy response in TNBC.

## RESULTS

### TBXA2R expression is important for TNBC cell viability

**Figure 1:**
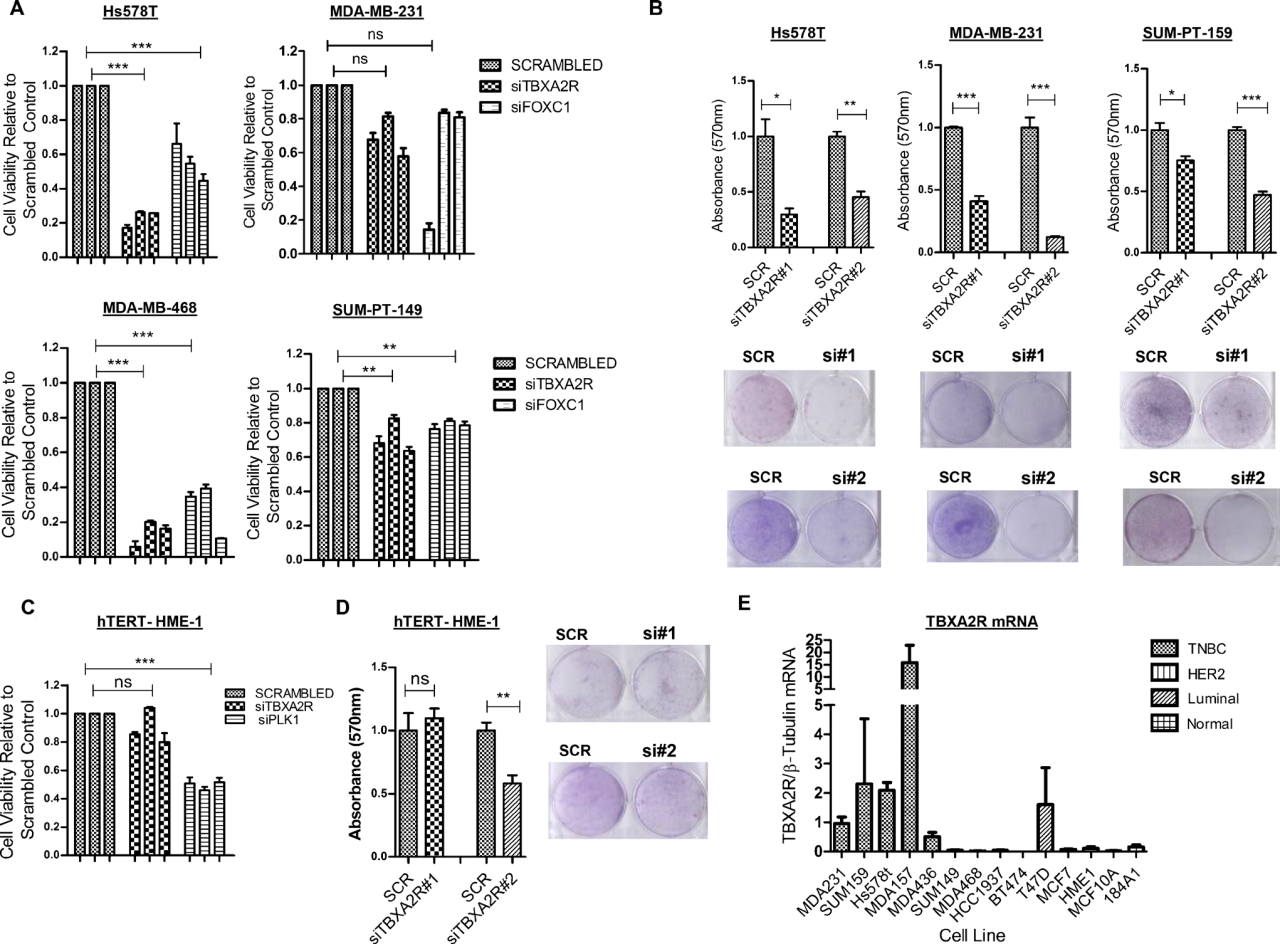
TBXA2R is a basal-specific marker and promotes survival of TNBC cells (**A**) Cell viability (as measured by MTT assay) following transfection of 3 independent TBXA2R siRNAs relative to scrambled siRNA control in the TNBC cell lines Hs578T, MDA-MB-231, MDA-MB-468 and SUM-PT-149 with FOXC1 siRNA was used as a positive transfection control. (**B**) Graphs showing absorbance values for crystal violet staining 72 hr post-transfection with scrambled siRNA control, TBXA2R siRNA#1 and #2 in Hs578T, MDA-MB-231 and SUM-PT-159 cells (representative clonogenic images underneath each graph). (**C**) Cell viability (as measured by MTT assay) following the same TBXA2R siRNA knockdowns as in (A) in the non-tumourigenic hTERT-HME1 cell line with (**D**) the corresponding TBXA2R knockdowns and crystal violet absorption as outlined in (B). Absorbance values following reabsorption of crystal violet represent results from 3 biological replicates. Data was analysed by one-way ANOVA with Dunnett's *post-hoc* test where *P < 0.05, **P < 0.01, ***P < 0.001 and ns = not significant. (**E**) qPCR analysis of TBXA2R mRNA levels in a panel of breast cancer and normal breast cell lines, normalised to β-tubulin mRNA levels.

An siRNA library screening approach was employed to measure effects on cell viability in TNBC cell lines following siRNA knockdown (using 3 independent siRNA sequences) of a number of genes differentially expressed in ‘good’ versus ‘poor’ outcome TNBC profiles (Supplementary Figure S1). Substantial reductions in cell viability as measured by MTT assay were observed following siRNA knockdown of multiple genes (relative to scrambled siRNA control) with pronounced viability effects observed with TBXA2R depletion in all 4 TNBC lines (MDA-MB-231, Hs578T, MDA-MB-468 and SUM-PT-149; Figure 1A). Triplicate knockdowns with two additional independent siRNAs, followed by crystal violet staining (to quantify cell density) again showed that depletion of TBXA2R reduced the viability of TNBC cell lines (Figure 1B). Conversely, minimal effects on cell proliferation were observed following reduction of TBXA2R in the non-tumorigenic basal breast line hTERT-HME-1 by both MTT assay (Figure 1C) and crystal violet staining (Figure 1D). TBXA2R mRNA expression was then measured in a panel of breast cell lines by quantitative real time PCR (qPCR), showing that TBXA2R expression is specifically elevated in TNBC cell lines relative to non-tumorigenic breast, HER2-overexpressing or luminal breast cancer lines (Figure 1E).

### TBXA2R is transcriptionally repressed by BRCA1

**Figure 2:**
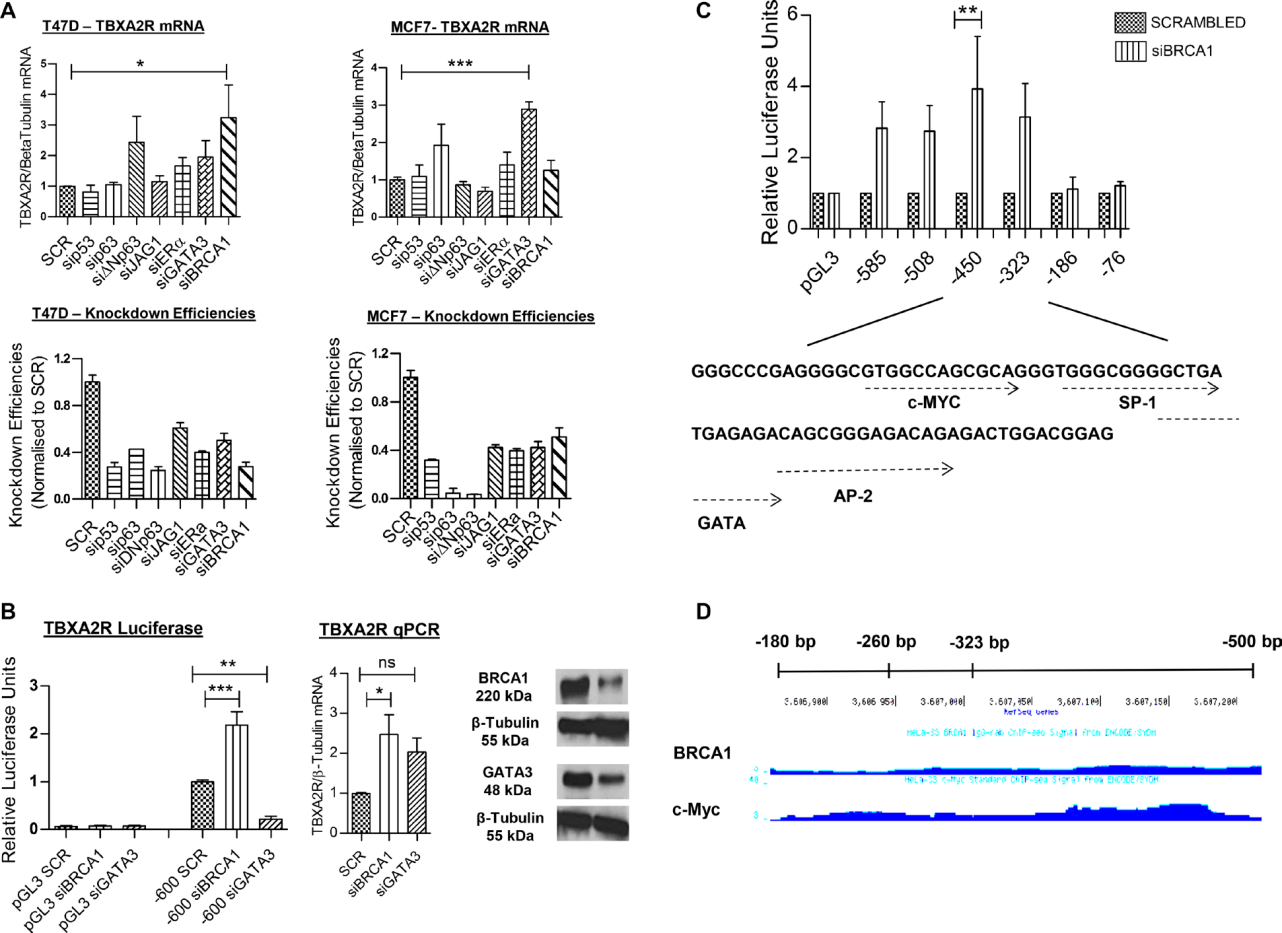
TBXA2R is negatively regulated by BRCA1 (**A**) qPCR analysis of TBXA2R mRNA levels normalised to β-tubulin mRNA in T47D and MCF7 cell lines following siRNA knockdown of p53, p63, ΔNp63, JAG1, ERα, GATA3 and BRCA1. Knockdown efficiencies are also shown for each gene immediately underneath. (**B**) Luciferase reporter gene assay of TBXA2R promoter region (−600 bp upstream of the Transcriptional Start Site, TSS) relative to pGL3 empty vector, following siRNA knockdown of BRCA1 and GATA3 in T47D cells. Matched qPCR shows TBXA2R mRNA levels 72 hr post-transfection of BRCA1 and GATA3 siRNA, and western blot analysis confirmed depletion of BRCA1 and GATA3 protein levels. (**C**) Luciferase reporter assay of T47D cells transfected with sequentially shorter TBXA2R promoter constructs following BRCA1 depletion, and underneath shows potential transcription factor binding sites within this region as predicted by the Alibaba online resource. Data represents 3 independent replicates analysed by one-way ANOVA with Dunnett's (A) or Bonferroni's (B, C) post-hoc tests; *P < 0.05, **P < 0.01, ***P < 0.001 and ns = not significant. (**D**) ENCODE analysis of BRCA1 and c-Myc binding on TBXA2R promoter region of interest in HeLa cells.

To identify the mechanism of transcriptional regulation of TBXA2R in breast cancer cells, a number of key transcription factors were depleted by siRNA in T47D and MCF7 cell lines and changes in TBXA2R mRNA levels were measured by qPCR. These two breast cancer lines were chosen since they are known to express wild-type BRCA1 and luminal differentiation markers (some of which we targeted here). Figure 2A shows a statistically significant increase in TBXA2R gene expression following siRNA knockdown of BRCA1 in T47D cells and GATA3 in MCF7 cells. This is an interesting observation since our group have previously shown that BRCA1 and GATA3 interact to co-repress another basally-restricted oncogene, FOXC1 [24]. Efficiencies of siRNA knockdowns were comparable in both cell lines (shown immediately underneath). Luciferase reporter assays were carried out in T47D cells following transfection of a proximal region of the TBXA2R promoter (600 bp upstream of the transcriptional start site, TSS). Following depletion of BRCA1, luciferase activity was significantly increased indicative of enhanced TBXA2R promoter activity (Figure 2B) in agreement with TBXA2R gene expression (Figure 2A and 2B). However, in contrast to increased TBXA2R mRNA following reduction of GATA3, TBXA2R promoter activity was reduced suggesting that GATA3 is not acting as a co-repressor with BRCA1 within this proximal promoter region with western blot analysis confirming knockdown of both BRCA1 and GATA3 at the protein level (Figure 2B). To define the mechanism of BRCA1-mediated repression of TBXA2R, we used exonuclease mapping to generate sequentially shorter TBXA2R promoter luciferase reporter constructs to assess transcriptional activity following transfection of BRCA1 siRNA. Figure 2C shows BRCA1 regulation is lost between −323 and −186 bp relative to the Transcriptional Start Site (TSS) of TBXA2R. As BRCA1 cannot bind DNA in a sequence specific manner, we used TF Search and Alibaba online resources to predict potential transcriptional regulators. These showed potential binding sites for a number of known BRCA1-interactors including SP-1, AP2, GATA family and c-Myc (Figure 2C). ENCODE analyses demonstrated ChIP-seq experiments which showed potential overlapping binding regions for BRCA1 and c-Myc on the TBXA2R promoter in HeLa cells (Figure 2D).

### c-Myc and BRCA1 act as co-repressors of TBXA2R transcription

**Figure 3:**
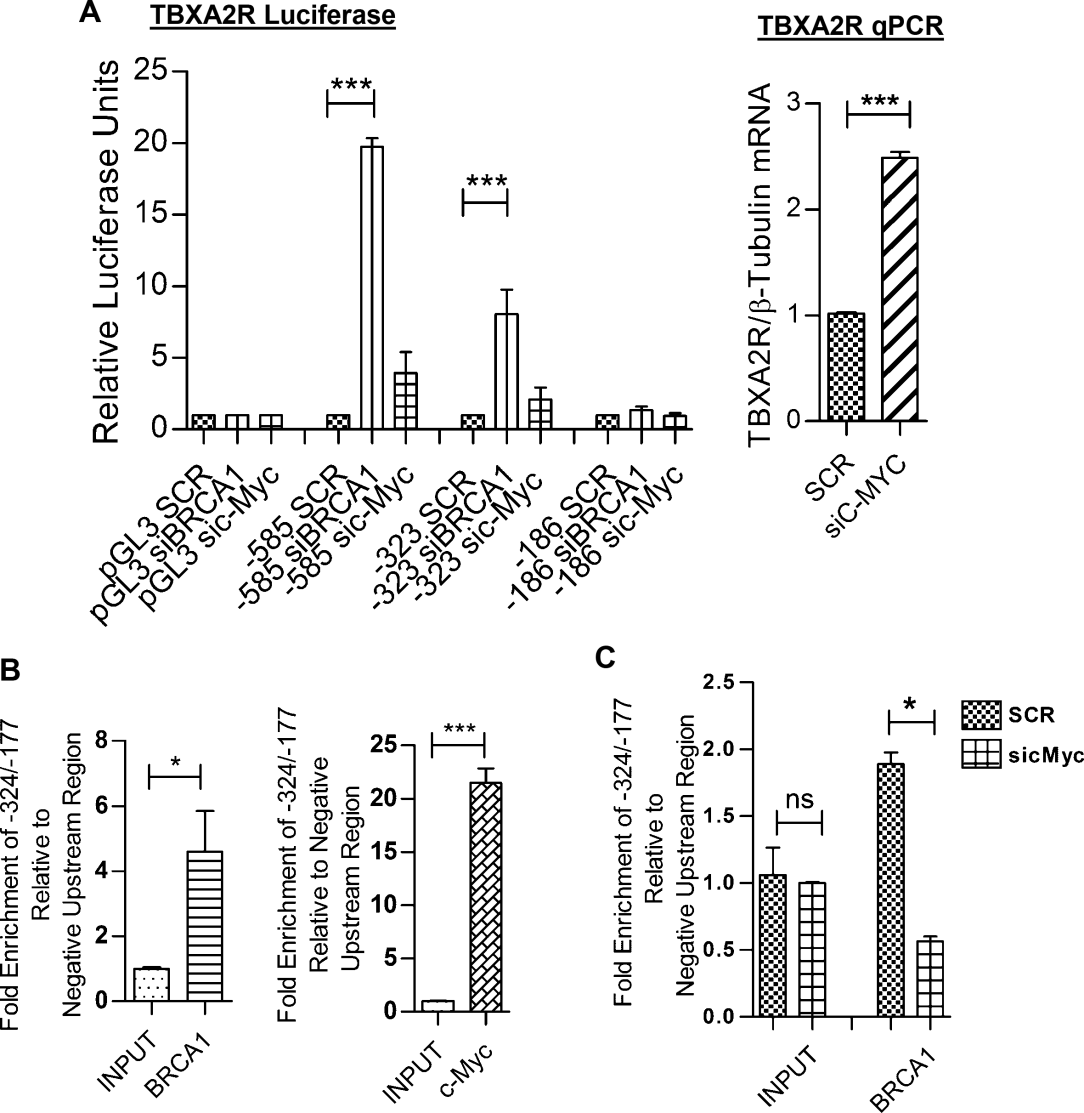
BRCA1 is recruited to the TBXA2R promoter by c-Myc (**A**) Luciferase reporter assays of TBXA2R promoter constructs (−585, −323 and −186 bp upstream of the TSS) 72 hr post-transfection with BRCA1 and c-Myc siRNA. Data normalised to pGL3 Basic and scrambled siRNA control (*n* = 3). (**B**) qPCR analysis of TBXA2R mRNA levels following siRNA knockdown of c-Myc (with mRNA values normalised to β-tubulin mRNA). (**C**) ChIP assay in T47D cells showing fold enrichment of BRCA1 and (**D**) c-Myc binding to the TBXA2R promoter relative to a negative upstream region as analysed by qPCR. Data was analysed by unpaired one-tailed *t*-test (*n* = 3); *P < 0.05, ***P < 0.001. (**E**) ChIP assay showing differential recruitment of BRCA1 to the TBXA2R promoter following siRNA depletion of c-Myc. ChIP data was normalised to chromatin input. Data was analysed by one-way ANOVA and Bonferroni's post-hoc test; *P < 0.05 and ns = not significant; *n* = 4 replicates over two independent experiments.

C-Myc has been well documented as a BRCA1 interacting protein and an important protein for the ability of BRCA1 to transcriptionally repress basal-like genes such as psoriasin and p-cadherin [25, 26]. Physical interaction between BRCA1 and c-Myc has also been shown specifically in T47D cells [26], therefore, we hypothesised this may be a likely mechanism through which TBXA2R is repressed. In agreement with this, Figure 3A shows TBXA2R promoter activity was increased at −585 bp and −323 bp following transfection of c-Myc siRNA and regulation is lost at −186 bp upstream of the TSS. This followed the same trend as BRCA1 knockdown (although c-Myc siRNA did not reach statistical significance). Figure 3B shows increased TBXA2R mRNA in T47D cells following depletion of c-Myc. BRCA1 and c-Myc binding to the TBXA2R promoter was confirmed by chromatin immunoprecipitation (ChIP) assay. Figure 3C and 3D show enrichment on the −324/−177 bp region (as analysed by qPCR) of the TBXA2R promoter (relative to a negative upstream region) following immunoprecipitation of chromatin using both BRCA1 and c-Myc antibodies respectively, demonstrating that both these proteins are bound to this region of the promoter. Indeed, qPCR of immunoprecipitated DNA showed that following c-Myc depletion BRCA1 could not be localised to the TBXA2R promoter region, indicating that c-Myc is required for BRCA1 recruitment (Figure 3E).

### TBXA2R signals through the Rho/ROCK pathway in TNBC cells

**Figure 4:**
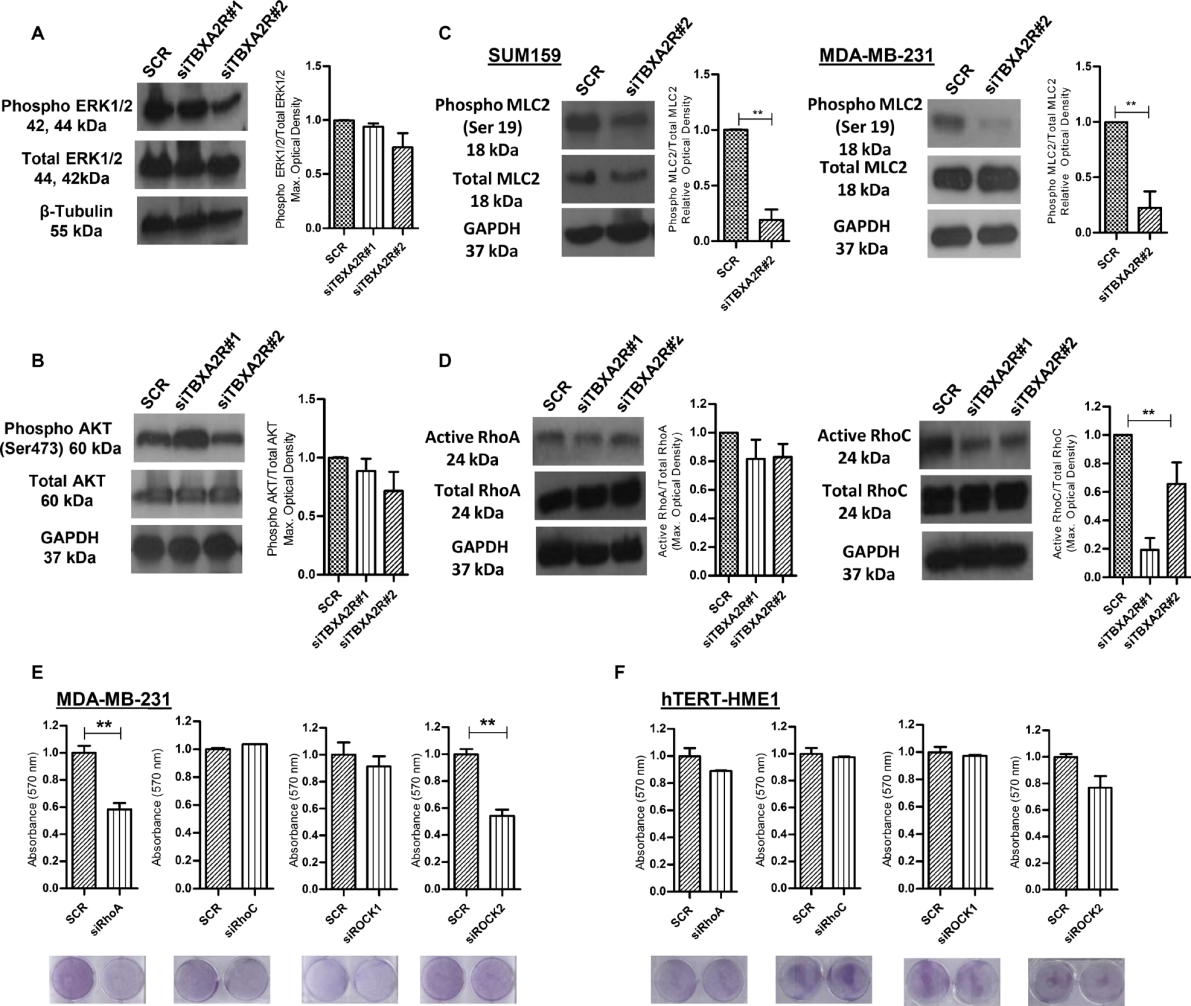
ROCK/Rho pathway is activated by TBXA2R (**A**) Western blot analysis of total and phosphorylated ERK1/2 in MDA-MB-231 and (**B**) total and phosphorylated AKT (Ser 473) levels in SUM-PT-149 cells 72 hr post-transfection of 2 independent TBXA2R siRNAs. Densitometry values were normalised firstly for total ERK to β-tubulin and then phosphorylated values were normalised against total ERK/β-tubulin. (**C**) Western blot analysis of total and phosphorylated MLC2 (Ser 19) levels in SUM-PT-159 and MDA-MB-231 cells 72 hr post-transfection with 2 independent TBXA2R siRNAs with accompanying graphs showing densitometry values for phospho-MLC2 (Ser 19) relative to total MLC2 levels in SUM-PT-159 cells. (**D**) Western blot analysis of GTP-RhoA and GTP-RhoC levels were performed in the same two TNBC cell lines 72 hr post-transfection with 2 independent TBXA2R siRNAs, with accompanying graphs showing densitometry values for active Rho relative to total Rho levels. GAPDH was used as a loading control in (A–D) and blots were quantified by densitometry of 3 independent replicates. (**E**) Graphs showing absorbance values (*n* = 3) for crystal violet staining of MDA-MB-231 and (**F**) hTERT HME-1 cells 72 hr post-transfection with RhoA, RhoC, ROCK1 and ROCK2 siRNAs with representative images of plates underneath each graph. Data was analysed by one-way ANOVA with Dunnett's post-hoc test (A, B, D) or unpaired two-tailed *t*-test (C, E, F) ; **P < 0.01.

The molecular mechanisms by which TBXA2R maintains cell viability in TNBC are currently undefined. Previous studies have shown TBXA2R can regulate a number of pathways involved in oncogenesis, most notably through the MAPK/ERK, AKT and Rho pathways [14, 15, 21, 27]. Western blot analysis shows minimal variation in levels of phosphorylated ERK1/2 in MDA-MB-231 (Figure 4A) or levels of phosphorylated AKT(Ser473) in SUM-PT-159 cells (Figure 4B) following independent TBXA2R siRNA knockdowns. Depletion of TBXA2R leads to significantly reduced levels of phosphorylated myosin light chain 2 (pMLC2) in both MDA-MB-231 cells and SUM-PT-159 cells (Figure 4C). MLC2 is known to be phosphorylated (and activated) downstream of Rho/ROCK and has been used as a reliable surrogate marker of Rho pathway activation. To confirm the involvement of the Rho pathway, direct Rho activation assays were carried out in which a GST-Rhotekin binding domain fusion protein was used to specifically immunoprecipitate the active GTP-bound form of Rho. Levels of active Rho A and C isoforms analysed by western blot show decreased levels of both active RhoA and C following TBXA2R knockdown (Figure 4D). However, only reductions in levels of RhoC were statistically significant. To show which members of the Rho pathway effect cell viability (in a manner similar to TBXA2R) individual members of the Rho pathway were knocked down by siRNA and cell viabilities were analysed by crystal violet staining. Specifically, RhoA and ROCK2 knockdowns both significantly decreased cell viability in MDA-MB-231 cells (Figure 4E) but showed minimal effects on ‘normal’ hTERT-HME-1 cell viability (Figure 4F), recapitulating the previously shown TBXA2R siRNA effects.

To unambiguously confirm that the phenotypic effects that we have observed where wholly due to TBXA2R and not to non-specific targeting via siRNA knockdowns, we performed a rescue experiment. Here we stably expressed exogenous TBXA2R in a TNBC cell line and then knocked down endogenous TBXA2R with an siRNA oligonucleotide (designed against the 5′UTR region of TBXA2R mRNA in order to target only the endogenous mRNA) prior to performing an array of phenotypic readouts. As Supplementary Figure S4 shows, we could demonstrate that expression of exogenous TBXA2R back into TBXA2R 5′UTR siRNA depleted MDA-MB-231 cells (exogenous TBXA2R qPCR shown in (a)) totally rescued cell viability (b), cell migration (c) (assessed by wound closure) and activation of Rho signalling (d) (assessed by phospho-MLC2 immunoblotting and quantified by densitometry relative to total MLC2, Figure 4E). These data together categorically demonstrate that the TBXA2R signalling pathway is a key driver of multiple oncogenic phenotypes in TNBC cells.

### ROCK inhibition decreases cell viability, migration and invasion in TBXA2R-expressing TNBCs

**Figure 5:**
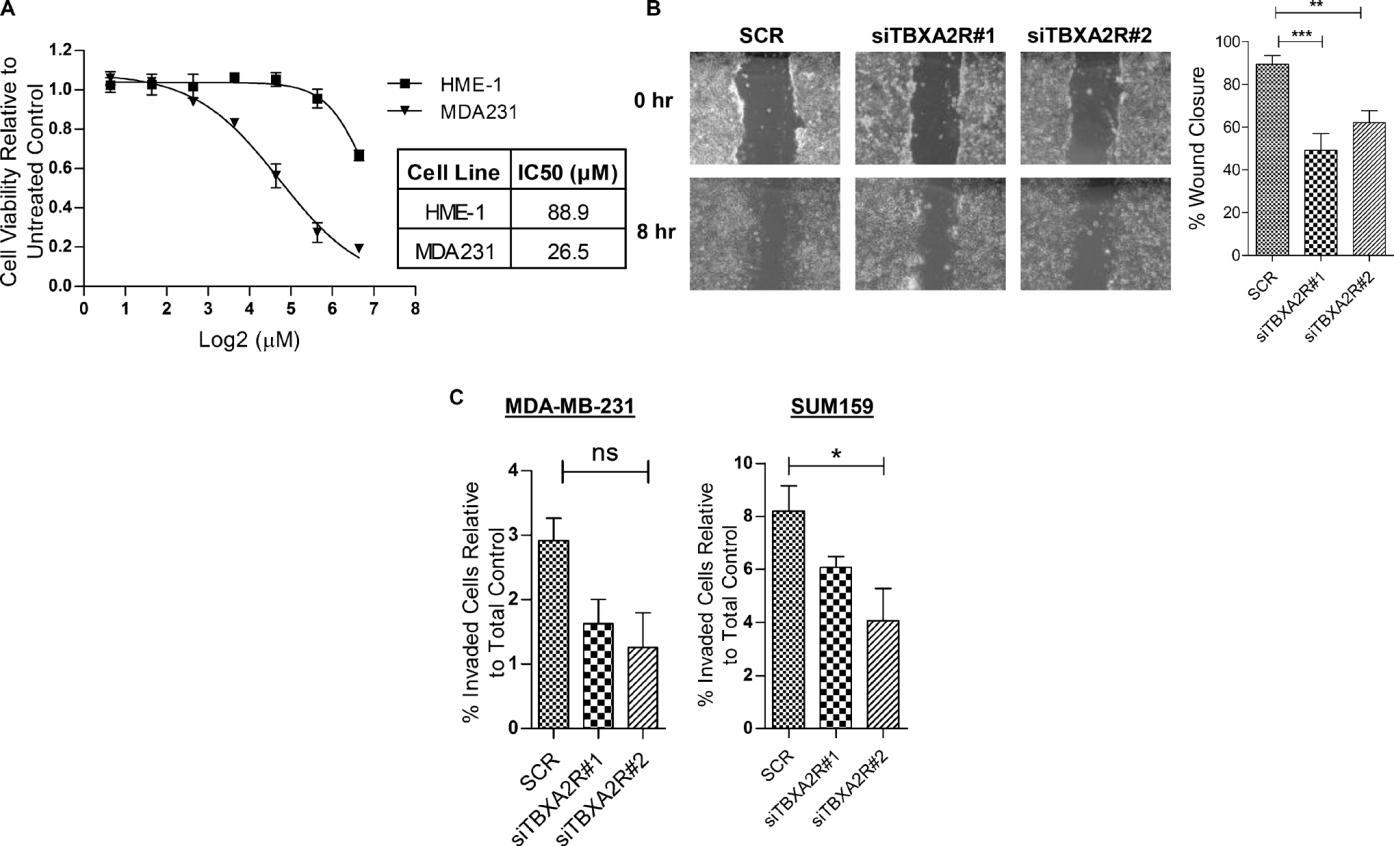
ROCK inhibitors as a potential therapeutic strategy for TBXA2R-expressing TNBCs (**A**) Dose-response curve showing cell viabilities (as analysed by MTT assay) of hTERT-HME1 and MDA-MB-231 cells following 48 hr treatment with a range of concentrations of the ROCK inhibitor Y39983. IC50 values for both cell lines are shown. (**B**) Micrographs of wound scratch experiments at 0 hr and 8 hr in Hs578T cells, 48 hr post-transfection with scrambled siRNA and 2 independent TBXA2R siRNAs. Graph showing quantification of wound closure is also shown on right (*n* = 3), analysed by one way ANOVA with Dunnett's post-hoc test; **P < 0.01, ***P < 0.001, ns = not significant. (**C**) Matrigel-coated Boyden chamber invasion assays in MDA-MB-231 and SUM-PT-159 cells showing the percentage of cells invaded relative to the total number of cells, following transfection with scrambled and two independent TBXA2R siRNAs. Data represents 3 independent replicates and was analysed by one way ANOVA with Dunnett's post-hoc test; *P < 0.05.

Since the knockdown studies from Figure 4 show that ROCK activity may maintain cell viability of TNBCs downstream of TBXA2R we wanted to assess the effects of ROCK inhibitors. A cell viability graph following treatment with ROCK inhibitor Y39983 demonstrated a lower IC50 value for MDA-MB-231 TNBC cells compared to hTERT-HME-1 (Figure 5A), suggesting a potential therapeutic window for ROCK inhibitor treatment of TNBCs. In addition to our observations on cell viability, previous studies have also reported TBXA2R to augment cell migration and invasion [17, 18]. Wound scratch assays demonstrated that TBXA2R depletion decreased cell migration (Figure 5B), whilst Matrigel-coated Boyden chamber assays showed reductions in cell invasion in both MDA-MB-231 and SUM-PT-159 cells (Figure 5C).

### TBXA2R antagonises endogenous ROS levels in TNBC cell lines and is a potential biomarker of TNBC clinical outcomes

**Figure 6:**
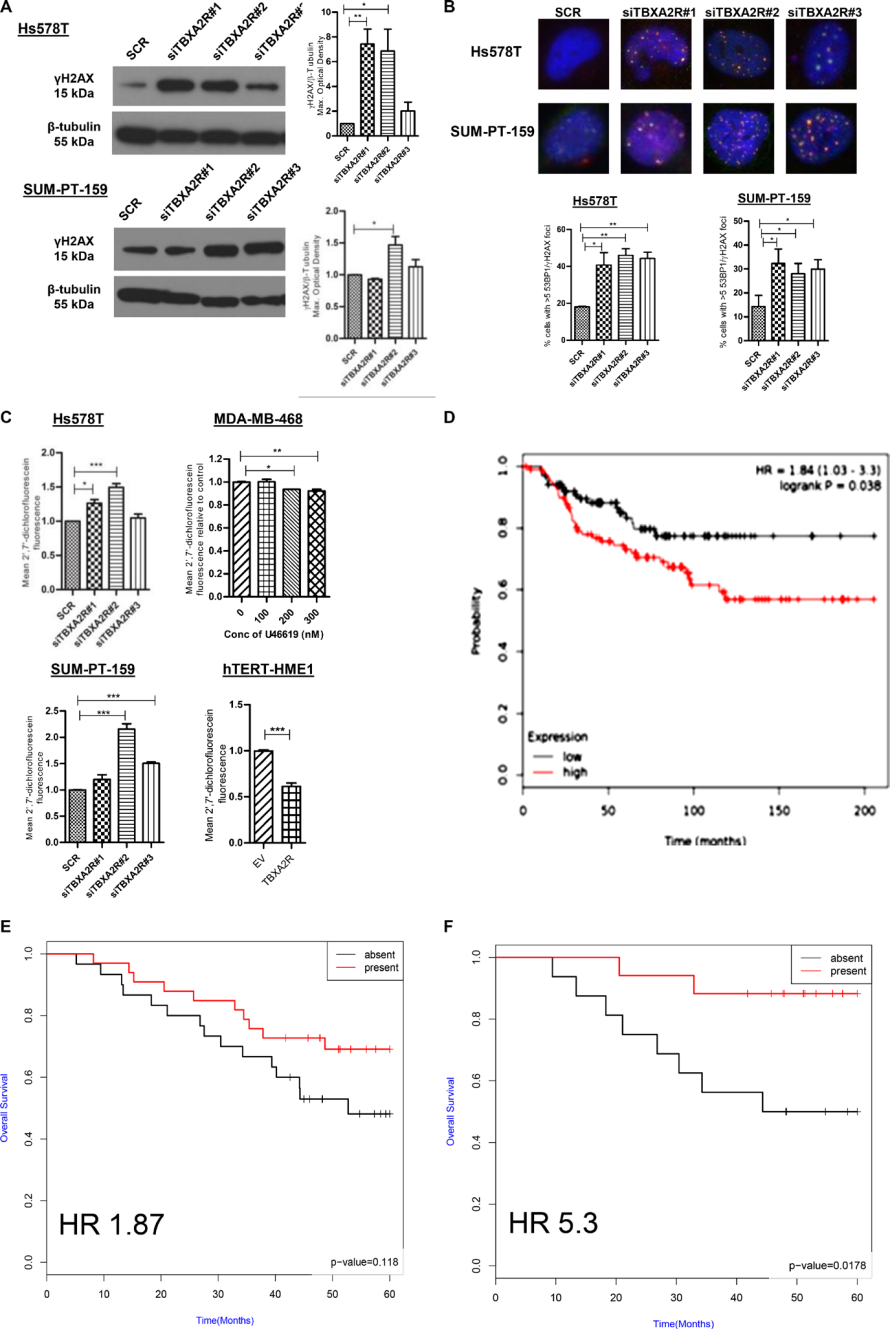
TBXA2R knockdown induces markers of DNA damage and ROS in TNBCs with TBXA2R expression correlating with TNBC clinical outcomes (**A**) Western blot analysis of γH2AX levels in Hs578T and SUM-PT-159 cells 72 hr post-transfection with 3 independent TBXA2R siRNAs or scrambled siRNA control. β-tubulin was used as a loading control. Graphs on right show γH2AX levels from 3 biological replicates quantified by densitometry and analysed by one way ANOVA with Dunnett's post-hoc test; *P < 0.05, **P < 0.01. (**B**) Merged images of immunofluorescent staining of Hs578T and SUM-PT-159 with γH2AX (red) and 53BP1 (green) antibodies following transfection of 3 independent TBXA2R siRNAs (60x magnification). Foci were counted in > 100 cells from 4 different fields and percentage of cells with > 5 foci was calculated. Data is representative of 3 independent experiments analysed by one way ANOVA with Dunnett's post-hoc test; *P < 0.05, **P < 0.01. (**C**) Flow cytometry analysis of fluorescent DCF formed by oxidation of non-fluorescent carboxy-2′7′-dichlorofluorescein (H2DCF) substrate to quantify ROS levels. ROS assay was carried out 48 hr post-transfection with 3 independent TBXA2R siRNAs in Hs578T and SUM-PT-159 cells, 1 hr post-treatment with U46619 (100, 200 or 300 nM) in MDA-MB-468 cells and 48 hr following over-expression of TBXA2R in HME-1 cells. (**D**) Kaplan-Meier curves from publically available metadata (KM Plotter) showing overall survival of unselected (all subtype) breast cancer patients. (**E**) Kaplan-Meier curves from IHC staining of an in-house tissue microarray of TNBC patients (*n* = 63) showing that increased TBXA2R expression correlates with Overall Survival (OS) of TNBCs (*p* = 0.118, HR 1.87). (**F**) Kaplan-Meier curves from IHC staining of the same in-house tissue microarray of TNBC patients (*n* = 63) (described in E), showing that increased TBXA2R expression correlates significantly with TNBC patients stratified for treatment with DNA damaging (FEC) chemotherapy (*p* = 0.0174, HR 5.3).

Since TBXA2R has well documented roles in oxidative stress responses we postulated that reducing TBXA2R levels may induce apoptosis *via* generation of intracellular oxidative stress via ROS. The thromboxane pathway has previously been shown to regulate levels of ROS which are a major endogenous source of DNA damage in multiple tissue types [28]. Levels of the early DNA damage marker γH2AX were measured by immunoblot in Hs578T and SUM-PT-159 cells which showed increased γH2AX in both cell lines (quantified by densitometry) following triplicate independent TBXA2R siRNA knockdowns (Figure 6A). The increase in DNA damage following TBXA2R knockdown was also demonstrated by foci counts following immunofluorescent staining for γH2AX and 53BP1 in both Hs578T and SUM-PT-159 cells (Figure 6B and Supplementary Figure S2). We also observed increased levels of ROS following TBXA2R siRNA knockdowns in both Hs578T and SUM-PT-159 cell lines (Figure 6C). In contrast, over-expression of TBXA2R in non-tumorigenic HME-1 cells led to decreased intracellular ROS levels and treatment with the TBXA2R agonist U46619 reduced ROS levels in a dose dependent manner (Figure 6C) suggesting TBXA2R levels may be important in promoting cell viability by counteracting endogenous ROS generation. TBXA2R clearly has a role in maintaining and driving pathogenic features of TNBC cell lines but we were also interested in testing its utility as a potential prognostic biomarker of clinical outcome, or as a predictive biomarker of DNA damaging chemotherapy responses in TNBC patients. Using publically available gene expression data we observed that TBXA2R (as predicted given that it is a basally restricted marker) represents a poor prognosis marker of breast cancer overall using KM plotter (Figure 6D, *p* = 0.038, HR 1.84). We internally validated immunohistochemical (IHC) staining for TBXA2R and performed IHC on an in-house tissue microarray containing 63 TNBC cases (3 tumour cores from each case) with 5 year clinical follow-up. Measuring Overall Survival as a clinical end-point, we observed a non-significant trend that increased TBXA2R expression was a predictor of good outcome in TNBCs (Figure 6E, *p*-value = 0.118, HR 1.87) but this trend became statistically significant only when FEC treated patients (*n* = 33) were included in the comparison (Figure 6F, *p*-value = 0.0178, HR 5.3). Similar results were observed using Relapse Free Survival as clinical end-point (Supplementary Figure S3A and S3B, respectively). Together these analyses indicate that TBXA2R may possess utility as a predictive marker of clinical outcomes in TNBC following FEC chemotherapy.

## DISCUSSION

In this study, we have identified TBXA2R as a novel survival factor for breast cancer which specifically maintains cell viability of TNBC cells. A substantial decrease in cell viability was observed following TBXA2R siRNA knockdowns in multiple TNBC cell lines, while lesser effects were observed on the growth of non-tumorigenic basal breast cell lines. We present evidence that (i) TBXA2R is transcriptionally repressed by a BRCA1/c-Myc complex, (ii) components of the Rho/ROCK pathway may be important mediators of TBXA2R signalling in TNBCs, (iii) TBXA2R may have roles in controlling endogenous ROS levels and DNA damage and (iv) whilst this receptor is a biomarker of poor prognosis in breast cancer overall, it may possess potential to serve as a predictive marker of good outcomes to DNA damage therapies in TNBCs. So whilst TBXA2R may be expressed at higher levels in good outcome TNBCs and represent a survival factor for this subpopulation of TNBCs, its expression may also be a useful marker of which TNBCs possess an underlying DNA repair defect.

TBXA2R can activate a diverse range of signalling pathways and has been implicated in the pathogenesis of many diseases, including cancer. Consistent with our findings that TBXA2R is highly expressed in a number of breast tumours, Watkins *et al.* have shown that TBXA2R mRNA is preferentially expressed in breast tumours in comparison to normal breast tissue, and a separate study showed TBXA2R gene expression was elevated in inflammatory breast cancer, however, the functional significance of TBXA2R in breast cancer has not been explored [23, 29].

The TBXA2R promoter has been mapped and Prm1 is the major promoter for transcriptional regulation of the predominant TBXA2Rα isoform [30, 31]. However, this is the first report that BRCA1 is a transcriptional repressor of TBXA2R. Our results suggest BRCA1 and c-Myc act as negative regulators of TBXA2R transcription by forming a functional transcriptional repressor complex on the TBXA2R promoter. Previous studies have shown the physical interaction of BRCA1 and c-Myc by both the yeast-2-hybrid method [32] and by co-immunoprecipitation in T47D [26], and the BRCA1/c-Myc complex has been shown to repress transcription at the promoters of a number of genes including CDC25A and hTERT [32, 33]. It is also interesting that BRCA1/c-Myc has been shown to repress transcription of p-Cadherin and psoriasin genes, both of which are preferentially expressed within the basal phenotype [25, 26]. Since BRCA1 is commonly inactivated in BLBC [34] it is perhaps not surprising that a number of basal markers including TBXA2R are negatively regulated by BRCA1. We suggest that the emergence of aggressive phenotypes observed following loss of BRCA1 function in breast cancer cells [35] may be partly due to the de-repression of TBXA2R. In contrast to BRCA1, c-Myc is often amplified in TNBC/BLBCs and has been particularly associated with loss of functional BRCA1 [36], therefore, further investigation is required into the effect of c-Myc on TBXA2R transcription in the absence of BRCA1. However, it has been suggested that BRCA1 may exert tumour suppressive effects by repression of c-Myc activated genes and while the tricomplex of BRCA1/Nmi/c-Myc represses hTERT transcription, c-Myc alone transcriptionally activates hTERT. Therefore, loss of BRCA1 in basal breast cells may alter the transcriptional properties of c-Myc [33]. Accordingly, overexpression of BRCA1 increases c-Myc binding to promoters of c-Myc/BRCA1 repressed genes, indicating BRCA1 may stabilise c-Myc to these promoters [25, 33].

Since the major role of TBXA2R involves platelet activation, this raises the possibility that direct therapeutic targeting of the receptor as an anti-cancer strategy may compromise haemostasis and would potentially be a contraindication for patients with bleeding disorders. TBXA2R activation of Rho signalling coupled to TNBC-specific decreases in cell viabilities following knockdown of RhoA and ROCK1/2 indicate that ROCK inhibition may be a potential therapeutic intervention in TBXA2R over-expressing TNBC. Rho, a small GTPase downstream of Gα_12/13_, is one of the major TBXA2R-coupled G-protein families and activation of TBXA2R in platelets stimulates this pathway resulting in platelet shape change mediated via the phosphorylation of MLC [37]. There are 3 Rho isoforms but little evidence of Rho isoform-specific effectors, so the mechanisms by which the isoforms exert differential effects are unknown, but RhoA and C tend to exhibit oncogenic effects in a number of cancers whereas RhoB suppresses tumourigenesis [38]. In breast cancer, RhoA and RhoC are both over-expressed and knockdown of each isoform decreased cell proliferation, migration and invasion in TNBC cell line MDA-MB-231 [39]. A specific role for RhoC has also been demonstrated in a mouse mammary adenocarcinoma model in which RhoC depletion abolished occurrence of lung metastasis [40]. In the current study, we have shown RhoA has more pronounced effects on cell viability of TNBC cells (similar to TBXA2R), than RhoC. However, TBXA2R knockdown demonstrated a greater depletion of active RhoC than RhoA [40, 41]. Together, this raises the possibility that activation of both of these Rho proteins may be responsible for the increase in cell proliferation, migration and invasion of TNBC observed with TBXA2R, but further investigation is required to determine if TBXA2R mediates these individual effects via specific Rho members. While the Rho/ROCK pathway is a well-established mediator of cell migration, reports of the effects of the Rho/ROCK pathway on cell survival are conflicting, with ROCK mediating both pro- and anti-apoptotic functions depending on the cellular context. For example, several reports support a pro-apoptotic role for ROCK in endothelial cells and erythroblastic TF-1 cells [42, 43] and in direct contrast to our findings, Harenburg *et al.* have found that TBXA2R-mediated Rho/ROCK activation causes apoptosis of immature thymocytes [44]. On the other hand, ROCK inhibition in anaplastic thyroid cancer cells and in glioma cells increased caspase-dependent apoptosis, indicating ROCK can also act as an anti-apoptotic protein in certain cells [45, 46].

In terms of therapeutics, a number of effective small molecule ROCK inhibitors have been developed, and the ROCK inhibitor, Fasudil, is already in clinical use in Japan for the treatment of sub-arachnoid haemorrhage, which has established the safety of these compounds [47]. Furthermore, previous studies have shown the metastasis-promoting ability of the Rho/ROCK pathway [21, 48] and demonstrated that ROCK inhibitors can impede breast cancer progression *in vitro* [49]. However, ROCK inhibitors have not yet progressed to clinical trials as anti-cancer agents in the UK or US. The discovery of TBXA2R as a driver of the oncogenic effects of Rho/ROCK in TNBCs presents the novel possibility that TBXA2R could be used as a biomarker to predict which patients may respond to treatment with ROCK inhibitors. This is of utmost importance since many clinical trials have failed in recent years due to poor patient selection, particularly for treatment of poorly defined TNBCs [50, 51]. Whilst PARP inhibitors have shown clinical utility in BRCA1/BRCA2 mutant breast and ovarian cancers, the story is much less clear in the majority of TNBCs, where surrogate markers of BRCA1 deficiency have not yet been identified. Our data indicate that ROCK inhibitor treatment may be a potential therapeutic strategy in TBXA2R-expressing/BRCA1-low TNBC patients for whom no targeted therapies are currently available. The fact that TBXA2R is elevated in the context of BRCA1 deficiency and predicts for favourable responses preferentially to FEC treatment would suggest it may possess utility as a predictive marker of good outcome following DNA damaging therapies in TNBCs. Clearly much more work is needed to truly evaluate the utility of both ROCK inhibitors and TBXA2R biomarker applications in TNBC clinical management.

## MATERIALS AND METHODS

### Cell lines

All cell lines were maintained as previously described [52] and were stored at 37°C in a humidified incubator at an atmosphere of 5% carbon dioxide in air.

### RNA extraction, cDNA synthesis and qPCR

Isolation of RNA from cell lines was performed using phenol-based RNA STAT-60^™^ (Amsbio, Abingdon, UK) according to manufacturer's instructions. RNA was reverse transcribed using the Transcriptor First Strand cDNA Synthesis Kit (Roche, Burgess Hill, UK) according to manufacturer's directions. Quantitative real-time PCR (qPCR) was carried out on the LightCycler^®^ 480 (Roche) using Light Cycler^®^ 480 SYBR Green I Master kit (Roche) following manufacturer's recommendations. qPCR primer sequences are listed in Supplementary Table S1.

### siRNA transfections

siRNA was transfected into cells using either Lipofectamine^®^ RNAiMax (Invitrogen, Paisley, UK (reverse transfection)) or Oligofectamine^™^ (Invitrogen (forward transfection)) according to manufacturer's protocol. siRNA sequences are listed in Supplementary Table S2.

### TBXA2R rescue experiments

TBXA2R was PCR cloned into the BamHI site of the retroviral vector pBabePuro. The pBabePuro-TBXA2R and pBabePuro EV constructs were transfected into Phoenix-Ampho 293T cells and after 48 hours virus containing media were collected, filtered and overlaid onto MDA-MB-231 cells overnight. Following removal of the transduction media cells were placed in selection media (1 μg/ml Puromycin) alongside a non-infected, negative control plate and cultured for up to 2 weeks. The EV and TBXA2R stably expressing mixed cell populations were then verified by qPCR for exogenous TBXA2R expression using vector-insert qPCR primers (Supplementary Table S1) and used for the phenotypic assays shown in Supplementary Figure S4.

### Cell viability assays

To assess cell viability, cells seeded in 96-well plates were incubated until an appropriate time-point at which 10 μl MTT ((5 mg/ml) Sigma-Aldrich, Dorset, UK) was added per well and incubated at 37°C with 5% CO_2_ for 2 hr. After removing media, 100 μl DMSO (Sigma-Aldrich) was added per well. Similarly, at defined time-points cells grown in 6-well plates were washed with PBS and incubated with crystal violet at room temperature for 15 min. The crystal violet was then rinsed off, left to air dry and reabsorbed in 0.1 M sodium citrate. The absorbance values for both MTT and crystal violet were read at a wavelength of 570 nm using the Biotrak II Visible plate reader (Amersham, GE Healthcare, Buckinghamshire, UK).

### Luciferase reporter rssays (including exonuclease mapping)

Luciferase constructs were transfected into T47D using the GeneJuice transfection reagent (EMD Millipore, Darmstadt, Germany) according to manufacturer's recommendations. Constructs used include pGL3 Basic empty vector, Renilla expression construct, pGL3 containing TBXA2R 600 bp promoter construct and pGL3 vector containing shorter TBXA2R promoter constructs generated using the exonuclease mapping ERASE-A-BASE kit (Promega, Southampton, UK), according to manufacturer's directions. Luciferase activity was measured as previously described [24].

### Chromatin immunoprecipitation assay

Chromatin Immunoprecipitation (ChIP) assays were carried out as previously described [53]. Antibodies used for ChIP were BRCA1 (Ab4) from Calbiochem (Darmstadt, Germany) and c-Myc (N-262) from Santa Cruz Technology (CA, USA). ChIP primer sequences are listed in Supplementary Table S3.

### Immunofluorescence

Transfected cells were seeded onto coverslips and at 72 hr post-transfection were fixed with 4% PFA. Following permeabilisation using 0.4% Triton X-100 (Sigma) in PBS, cells were blocked in 3% BSA/PBS and then stained with γ-H2AX and 53BP1 (both Millipore) primary antibodies, followed by incubation with Alexafluor 488-conjugated goat anti-rabbit and Alexafluor 568-conjugated goat anti-mouse secondary antibodies (Invitrogen). Coverslips were then mounted onto glass slides using ProLong^®^ Gold antifade mountant with DAPI (Thermo-Scientific) and imaged on a Nikon Eclipse Ti microscope, under a 60× objective.

### Reactive oxygen species assays

Cells were plated at 3 × 10^5^ cells per 35 mm dish. The next day the media was removed and cells were harvested in 1 ml cell dissociation buffer (Gibco, Paisley, UK), then pelleted and washed with PBS. H_2_DCFDA (Invitrogen) was added to samples at a concentration of 10 μM and assay was carried out according to manufacturer's protocol. Fluorescence of cell suspensions was analysed in the BD FACS Calibur flow cytometer.

### Western blot analysis

Immunoblotting was carried out as previously described [24]. For detection of phosphorylated proteins, cell lysates were collected in RIPA buffer (50 mM Tris pH 7.5, 150 mM NaCl, 1% NP-40, 0.5% sodium deoxycholate, 0.1% SDS) supplemented with phosphatase inhibitors: 1 mM sodium orthovanadate and 1 mM phenylmethanesulfonyl fluoride (PMSF; Sigma-Aldrich), as previously described [54]. Antibodies used include ERK, AKT, MLC2, pERK1/2, pAKT (Ser473), pMLC2 (Ser19) and RhoC (all Cell Signaling, MA, USA), γH2AX (Ser139) from Millipore and RhoA (26C4) from Santa Cruz.

### GST pull-down assay (Rho Activation)

The GST-Rhotekin Rho-binding domain (RBD) fusion protein (obtained from Prof. Chris Marshall, Cambridge University) was bound to prewashed Glutathione-coated Sepharose beads (GE Healthcare, Buckinghamshire, UK). A negative control was also set-up using GST-only ‘bait’ protein. Meanwhile, serum-starved cells were treated with TBXA2R agonist U46619 (Tocris, Abingdon, UK) at a concentration of 10 nm for 10 min and then lysed. The pre-washed GST-bound beads were then resuspended in 100 μl PBS to which 500 μg of cell lysate was added. This was rotated at 4°C for 30 min to allow binding of GST-Rhotekin RBD to GTP-Rho. The beads were pelleted and resuspended in protein sample buffer and levels of total Rho in whole cell lysate and active Rho in GST-Rhotekin RBD and GST-only pull-down samples were measured by Western blot analysis.

### Wound-scratch assay

Cells were seeded in 24-well plates at a density of 2 × 10^5^ per well. When confluent, a sterile yellow tip was used to make a vertical ‘wound’ in the centre of the well. The wounds were photographed at 10× magnification using the Olympus CKX41 microscope at 0 hr and at a suitable end time-point and the area of the wounds was measured using Cell^B software. The percentage wound closure was calculated by ((Area at 0 hr – area at end timepoint)/Area at 0 h)) × 100.

### Matrigel-coated boyden chamber assay

Millicell 12 μm cell culture inserts (Millipore) were coated with 60 μg BD Matrigel^™^ matrix (BD Biosciences, Oxford, UK) in serum-free media. The next day, cells were washed with serum-free media and 2.5 × 10^5^ cells per insert were seeded in serum-free media. To the well beneath the insert, 500 μl standard media containing 10% FCS was added. The cells were incubated at 37°C under 5% CO_2_ for approximately 22 hr. After this time, the membrane inside the inserts for analysing the invaded cells, but not for the ‘total cells’ control, were wiped with cotton wool. All inserts were fixed in methanol for 10 min and then stained with crystal violet for 30 min. After removing the crystal violet the inserts were left to dry and were then destained in 0.1 M sodium citrate and the absorbance was read at 570 nm using Biotrak II Visible plate reader. The percentage of invasive cells was calculated as follows: (absorbance of invaded cells/absorbance of ‘total cells’) × 100.

### Tissue microarrays (TMAs)

The breast cancer TMAs used in this study were constructed from formalin-fixed paraffin-embedded primary tumour blocks by the Northern Ireland Biobank (NIB ref: 12-0043) with each tumour sample represented by three independent 1 mm diameter cores. Repeat IHC for ER, PR and HER2 on the TMA sections confirmed the TNBC status of this case cohort. Anti-TBXA2R antibody was a kind gift from Therese Kinsella (UCD, Ireland). All antibodies were scored independently by two histopathologists blinded to patient clinicopathological and outcome data. TBXA2R was assessed and scored on the basis of presence or absence in tumour epithelium.

### Survival analyses

All Kaplan Meier Curves and Hazard Ratio Calculations were carried out using the R package “Survival”.

## SUPPLEMENTARY MATERIALS


