# Supplementary material for: Thromboxane A2 receptor (TBXA2R) is a potent survival factor for triple negative breast cancers (TNBCs)
Source: Oncotarget. 2016 Jul 30;7(34):55458–72. doi: 10.18632/oncotarget.10969 (PMC5342429; doi:10.18632/oncotarget.10969)
Supplement: Supplementary file 1 [file oncotarget-07-55458-s001.pdf]

Thromboxane A2 receptor (TBXA2R) is a potent survival factor for triple negative breast cancers (TNBCs)

Supplementary Materials

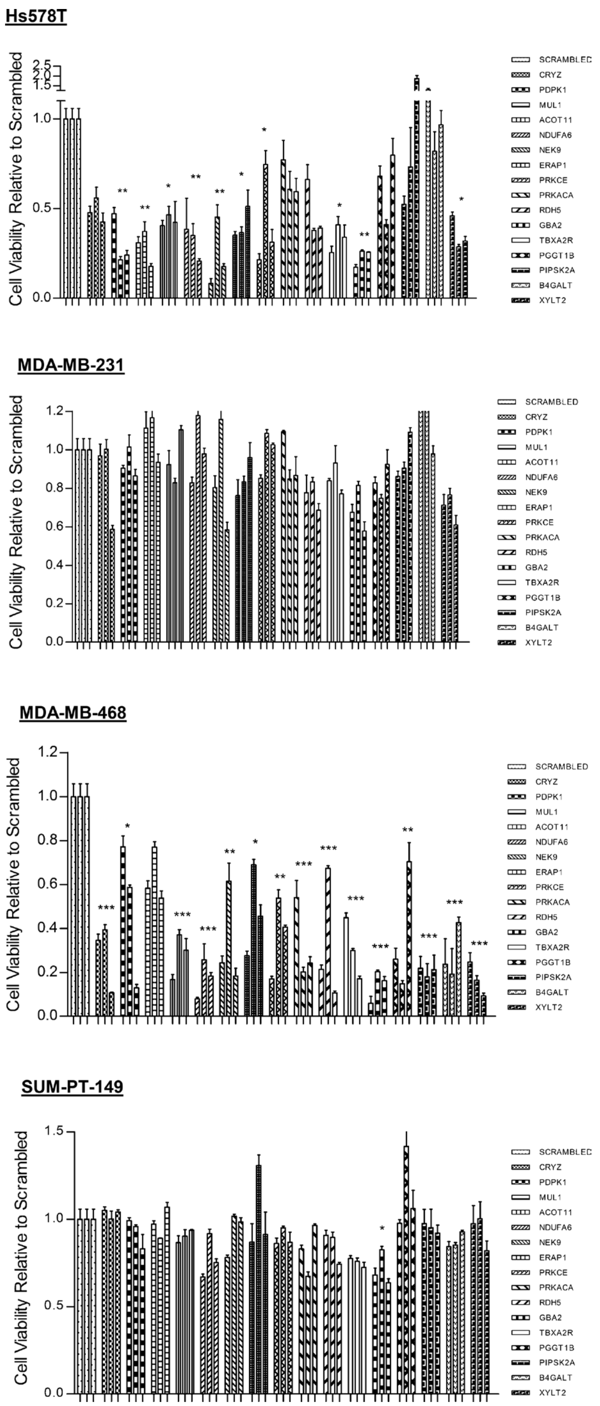

Supplementary Figure S1: siRNA library screens of genes differentially expressed in 'poor' versus 'good' outcome TNBCs. Cell viability measured by MTT assay following siRNA knockdown of a panel of genes normalised to scrambled siRNA control in Hs578T, MDA-MB-231, MDA-MB-468 and SUM-PT-149 cell lines. Statistical significance was calculated by One-Way ANOVA with Dunnett's Multiple Comparison test. * = $p < 0.05$, ** = $p < 0.01$, *** = $p < 0.001$, unmarked = not significant (Data represents 3 independent siRNAs against each gene and 3 technical replicates for each individual siRNA).

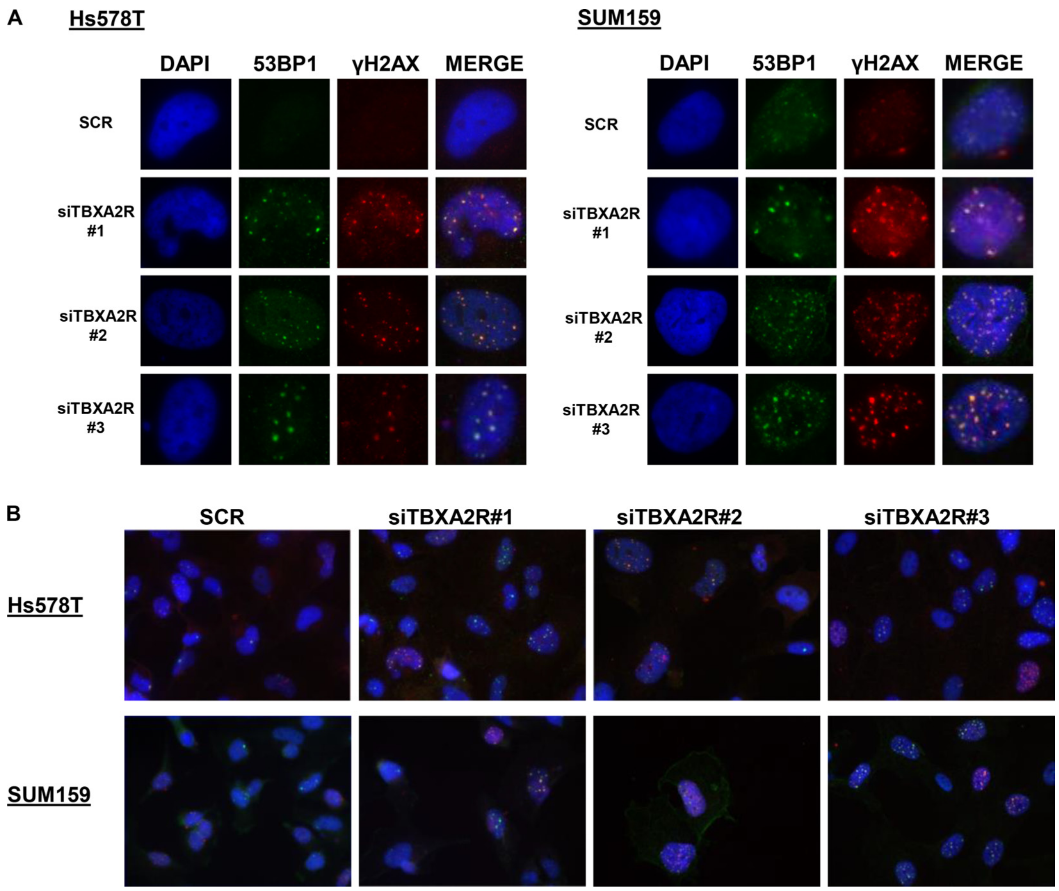

Supplementary Figure S2: Depletion of TBXA2R leads to increased levels of DNA damage. (**A**) Images of immunofluorescent staining of Hs578T and SUM-PT-159 (individual cells) following transfection of 3 independent TBXA2R siRNAs (60× magnification). From left: DAPI (blue), 53BP1 (green), γH2AX (red) and merged image. Data is representative of 3 independent experiments. (**B**) Merged images of immunofluorescent staining of Hs578T and SUM-PT-159 cells (selection of cells) with 53BP1 (green) and γH2AX (red) following transfection of 3 independent TBXA2R siRNAs (60× magnification).

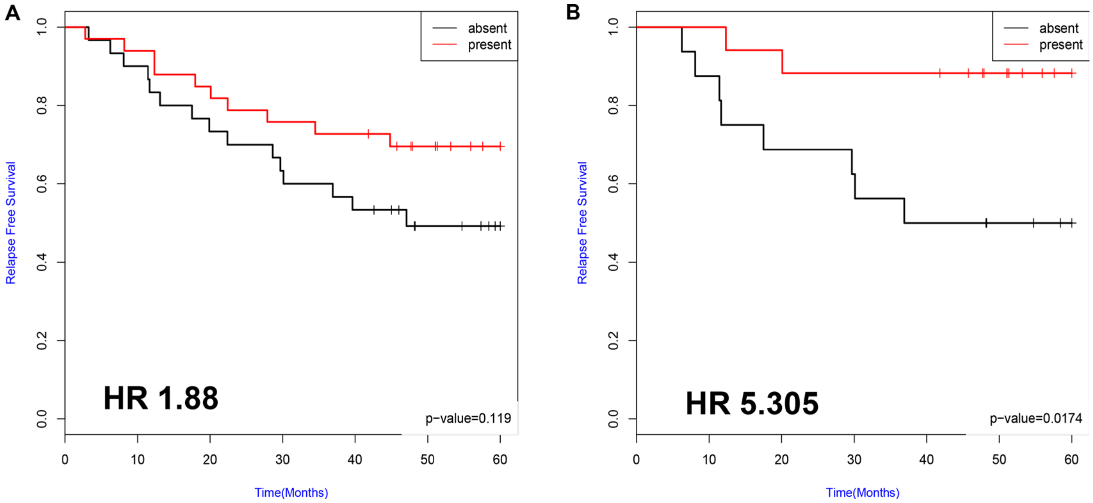

Supplementary Figure S3: TBXA2R is a predictive marker of Relapse-Free survival in TNBC patients. Kaplan-Meier curves from IHC staining of an in-house tissue microarray of TNBC patients ($n = 63$) showing that increased TBXA2R expression correlates with Relapse-Free Survival (RFS) of TNBCs ($p = 0.119$, HR 1.88) and significantly with TNBC patients stratified for treatment with DNA damaging (FEC) chemotherapy ($p = 0.0174$, HR 5.3).

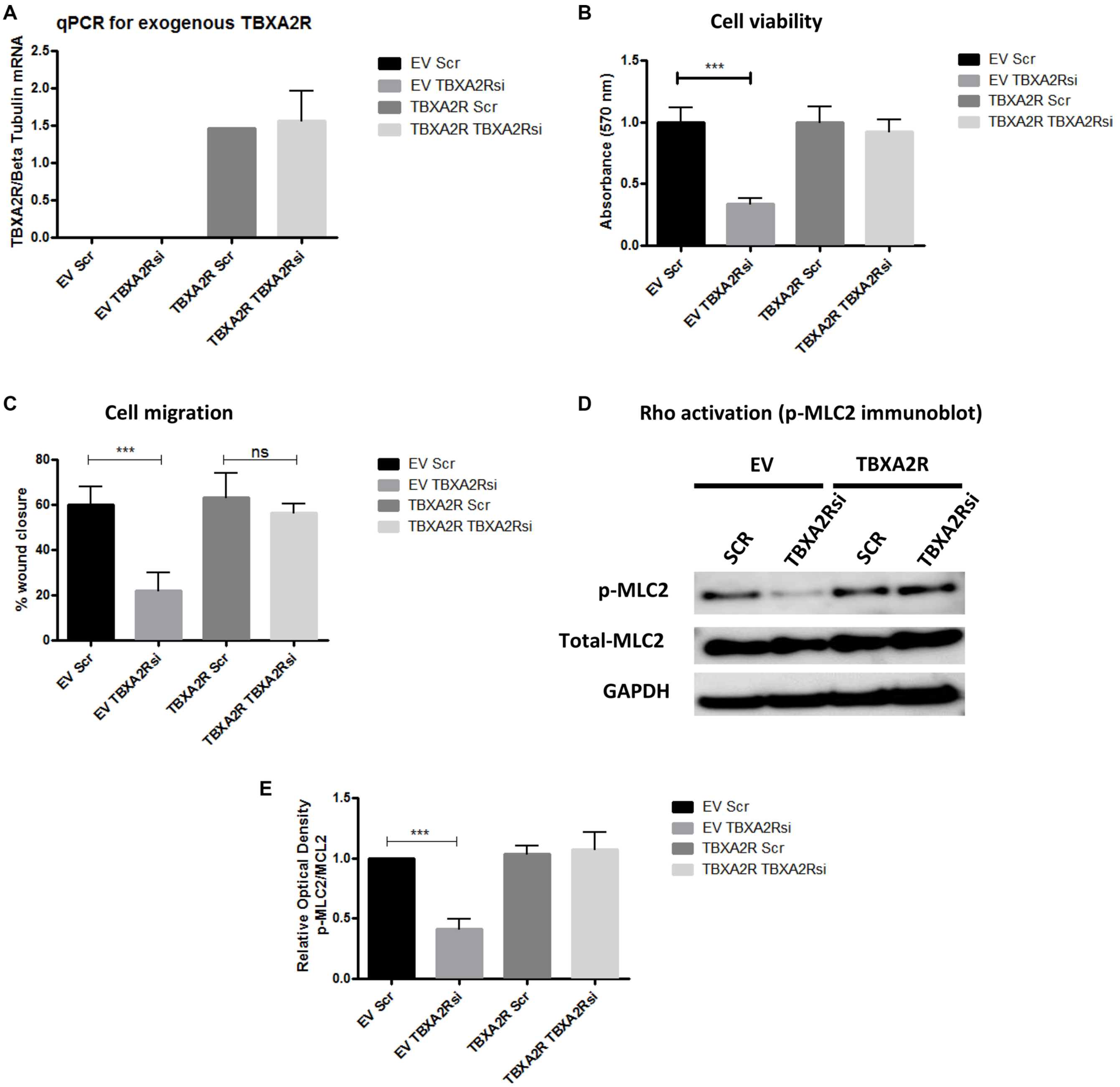

Supplementary Figure S4: Expression of exogenous TBXA2R (TBXA2R) rescues oncogenic phenotypes in TNBC cells depleted for endogenous TBXA2R (enTBXA2R). (**A**) Bar graph showing qPCR values for exogenous TBXA2R in EV and TBXA2R transduced MDA-MB-231 cells following treatment with scrambled (Scr) siRNA or an siRNA targeting endogenous TBXA2R siRNA (TBXA2Rsi). (**B**) Cell viability, (**C**) Cell migration and (**D**) phospho-MLC2 immunoblots in EV and exogenous TBXA2R transduced cells following treatment with scrambled siRNA (Scr) or an siRNA targeting endogenous TBXA2R siRNA (TBXA2Rsi). Cell viability and migration were both calculated relative to EV Scr (set at 1.0). (**E**) Densitometry of the immunoblot shown in (D) with the Relative Optical Density of phospho-MLC2 normalised relative to Total MLC2. In all bar graphs statistical significance was calculated by One-Way ANOVA with Dunnett's Multiple Comparison test. * = $p < 0.05$, ** = $p < 0.01$, *** = $p < 0.001$.

Supplementary Table S1: qPCR primer sequences

Primer		Sequence
β-Tubulin	F	CGCAGAAGAGGAGGAGGATT
	R	GAGGAAAGGGGCAGTTGAGT
TBXA2R	F	AGGTGGAGATGATGGCTCAG
	R	GTTTCGCAGCACTGTCTGG
p53	F	GCCTCACAACCTCCGTCATGT
	R	TCTGTCATCCAAATACTCCAC
p63	F	GACAGGAAGGCGGATGAAGATAG
	R	TGTTTCTGAAGTAAGTGCTGGTGC
ΔNp63	F	GTGGAATACGTCCAGGTGGC
	R	GGAAAACAATGCCCAGACTC
ERα	F	TGATGATTGGTCTCGTCTGG
	R	ATTTTCCCTGGTTCCTGTGG
GATA3	F	CAGACCACCACAACCACACTCT
	R	GGATGCCTCCTTCTTCATAGTCA
BRCA1	F	GGCTATCCTCTCAGAGTGACATTT
	R	GCTTTATCAGGTTATGTTGCATGG
JAG1	F	CGGGATTTGGTTAATGGTTAT
	R	ATAGTCACTGGCACGGTTGTAGCAC
TBXA2R-UTR for exogenous TBXA2R qPCR	F	GTGTATATCCTGTTCCGCCG
	R	CACTGGTCCAGGCACACCT

Supplementary Table S2: siRNA sequences

siRNA	Sequence
TBXA2R#1	CGGGCUGUCCUUCCUGCUGAAUU
TBXA2R#2	CCACAAACAUUACCCUGGA
TBXA2R#3	CCCGCAGAUGAGGUCUCUGUU
GFP-Scrambled	AAGCAGCACGACUUCUUCAAG
p53	UGUUCCGAGAGCUGAAUGA
p63	UUCCUCAGUCCAGAGGUUU
ΔNp63	GGACAGCAGCAUUGAUCAA
JAG1	CGCCAAAUCCUGUAAGAA
ERα	UCAUCGCAUUCCUUGCAAA
GATA3	AAGCCUAAACGCGUGGAUAU
BRCA1	GCGUGCAGCUGAGAGGCAU
c-Myc	CGACAGCAGCUCGCCCAAG
ROCK1	AAAUAGACAAGAGAUUACAGA
ROCK2	AACAAUAGAGAUCUACAAGAU
RhoA	AAGGCAGAGAUAUGGCAAACA
RhoC	AGAACUAUAUUGCGGACAU
TBXA2R-5UTR#2	CCU GAA CCA GUG CCA GCC U

Supplementary Table S3: ChIP primer sequences

Primer		Sequence
TBXA2R -1794/-1654	F	TATCTTTACCCCCTGCCTTG
	R	GTCCCCACTTCCTCTGTCCT
TBXA2R -324/-177	F	CAGAAGGCTGTAGGGTGTCC
	R	CTCGCTCTCTCCGTCCAGT
